# Longitudinal Trends of Comorbidities and Survival Among Kidney Cancer Patients in Asian Population

**DOI:** 10.1002/cam4.70421

**Published:** 2024-11-19

**Authors:** Minji Jung, Eunjung Choo, Jinhui Li, Zhengyi Deng, Marvin E. Langston, Sukhyang Lee, Benjamin I. Chung

**Affiliations:** ^1^ Department of Urology, School of Medicine Stanford University Stanford California USA; ^2^ Department of Clinical Pharmacy, School of Pharmacy Ajou University Suwon South Korea; ^3^ Department of Epidemiology and Population Health, School of Medicine Stanford University Stanford California USA

**Keywords:** comorbidity, kidney cancer, survivorship

## Abstract

**Background:**

Comorbidity could influence cancer diagnosis, treatment, prognosis, or survival. Although comorbidity burden in kidney cancer patients is high, limited evidence exists on the longitudinal patterns of individual comorbidity prevalence and its impact on overall survival among kidney cancer patients, particularly in Asian populations.

**Methods:**

We included adults diagnosed with kidney cancer between 2010 and 2021 using the Korean nationwide health insurance database. Comorbidities assessed were any 1 of 19 specific medical conditions, diagnosed within 1 year prior to cancer diagnosis. We calculated the incidence and age‐standardized incidence rate of kidney cancer, prevalence of individual medical conditions as single or multiple comorbidities, and overall survival probability of kidney cancer patients over a 12‐year period. We estimated the odds ratio (OR) of having individual and multiple comorbidities with age and sex as independent covariates and adjusted for other comorbidities. Kaplan–Meier curves were used for overall survival at different time frames up to 5 years of follow‐up.

**Results:**

Among kidney cancer patients (*N* = 42,740), 68.7% were men, and median (interquartile range) age was 59 (49–68) years. Approximately 76% of patients had at least one comorbidity at the time of cancer diagnosis. Overall, hypertension (51.3%), dyslipidemia (40.2%), mild liver disease (27.4%), diabetes (25.1%), and peptic ulcer disease (18.9%) were the most prevalent comorbidities. The proportion of patients having three or more comorbidities continuously increased from 2010 (29.4%) to 2021 (44.9%). Having more comorbidities was associated with a lower probability of overall survival.

**Conclusion:**

Comorbidities were prevalent in kidney cancer patients, and the proportions of patients with multiple conditions increased over time. Although survival probability increased over time, it was attenuated by having more comorbidities. Our data emphasizes the importance of comprehensive management for both cancer and comorbid conditions in kidney cancer patients.

## 1 Introduction

Kidney cancer survival has been rising for decades [1, 2, 3]. In 2023, the current 5‐year relative survival for all kidney cancer cases is 78.0%, increasing to 93.2% for localized cases, which accounts for nearly 80% of all cases [1]. This is largely due to significant advances in cancer treatments [4] and the increasing frequency of abdominal imaging investigations on non‐specific disorders, which may lead to incidental early detection [3].

In this growing population of kidney cancer patients with a longer life expectancy, the burden of comorbid conditions is high. Previous studies have shown that approximately half to three‐fourths of kidney cancer patients had one or more comorbidities, as indicated by the Charlson Comorbidity Index (CCI) score, a composite measure, at the time of cancer diagnosis [5, 6, 7, 8]. This could be attributed to various factors, including several shared risk factors (e.g., smoking, obesity, or hypertension) and common pathophysiological mechanisms (e.g., chronic inflammation, oxidative stress, or prothrombotic state) between kidney cancer and other medical conditions, such as chronic diseases or metabolic syndromes [9, 10]. Moreover, the aging population plays a role in the increasing prevalence of multiple comorbidities [11, 12].

Comorbidity status could impact cancer detection, diagnosis, treatment, prognosis, quality of life, or survival [5, 6, 7, 8, 13, 14, 15, 16]. However, current guidelines do not consider the complex interrelations between cancer and comorbidity but manage cancer as a single disease [4, 17]. To date, few studies have investigated the prevalence patterns of individual comorbidities among kidney cancer patients over time and their influence on overall survival [6, 7, 14, 18]. Rather than relying solely on a composite measure like CCI score, it is important to understand the comprehensive profile of individual comorbidities over time. Such an understanding would guide specific management strategies for different comorbidities among kidney cancer patients and provide insights into emerging trends that may require future attention. Our study sought to (1) estimate kidney cancer incidence over a recent 12‐year period (2010–2021); (2) identify the longitudinal prevalence patterns of individual comorbidities; and (3) examine their impacts on probability of overall survival, in an Asian population at a national level.

## 2 Method

### 2.1 Data Source

The Korean National Health Insurance Service (NHIS) program is a single‐payer, national health insurance program. The Korean NHIS covers over 97% of the entire Korean population, consisting of approximately 50 million people. In this nationwide population‐based study, we used the data acquired from the NHIS between 2007 and 2021 [19]. This data includes sociodemographic information, including age and sex, and clinical information, including diagnostic and prescription records from both inpatient and outpatient settings. Diagnosis was recorded according to the International Classification of Diseases, 10th Revision (ICD‐10) code. All individuals in the database were de‐identified, and this study was approved by the Institutional Review Board of Ajou University (IRB no.202209‐HB‐EX‐002).

### 2.2 Patient Identification

We identified individuals who were diagnosed with kidney cancer as a first primary cancer (ICD‐10 code, C64) between 2010 and 2021 (Figure S1). The diagnostic code was validated by V code (V193 and V194), which is a specific diagnostic code issued by the Korean NHIS. This helps verify cancer diagnoses through clinical and pathological assessments, consequently ensuring the accuracy of diagnostic codes [20]. The following individuals were excluded: (1) those aged under 20 years at the diagnosed date of kidney cancer and (2) those with a history of any cancers, except for non‐melanoma skin cancer, prior to kidney cancer diagnosis (Figure S2). All patients were followed until death or the end of the study period (December 31, 2021), whichever came first.

### 2.3 Measurements

Variables included age, sex, year of cancer diagnosis, and comorbidities. The age at cancer diagnosis was grouped into six categories: 20–39, 40–49, 50–59, 60–69, 70–79, and over 80 years. A total of 19 comorbidities were identified based on the CCI [21], listed in Table S1. Comorbidities were defined as at least one record of primary diagnosis or at least two separate records of diagnosis in either inpatient or outpatient settings within 1 year prior to cancer diagnosis. Among CCI groups, we excluded two groups (i.e., malignancy and metastatic carcinoma) because our study focuses on individuals diagnosed with kidney cancer as the first primary cancer. To reflect the substantial differences in risk of morbidity and mortality when a patient with chronic renal disease transitions to end‐stage renal disease, renal disease was divided into two groups: mild or moderate (chronic kidney disease, stage 1–4) and severe renal disease (stage 5). Additionally, five diseases were added using ICD‐10 codes, which might be associated with kidney cancer: hypertension, dyslipidemia, atrial fibrillation, chronic obstructive pulmonary disease (COPD), and obesity [22]. To assess the burden of single or multiple comorbid conditions, the number of comorbidities among the 19 diseases was calculated and classified into four groups: none, 1–2, 3–4, and 5 or more.

### 2.4 Descriptive and Statistical Analysis

The incidence of kidney cancer was estimated for each year of diagnosis, by age and sex. Age‐standardized incidence rate per 100,000 individuals (IR) by sex and year of diagnosis was adjusted to the 2020 Census from the Korean National Statistical Office [23]. Prevalence of individual comorbidity and the number of comorbidities were presented as the percentage of kidney cancer patients for age, sex, and each year of diagnosis. To investigate trends of comorbidity prevalence, if the 3‐year average is always higher or lower than the 3‐year average of the period immediately before, we identified it as an increased or decreased trend, respectively. To investigate the pattern of multiple comorbid conditions, relative frequency in ten of the most common conditions was estimated, either as a single or in combination with another comorbid condition. The relative frequency as the percentage was calculated as follows: the denominator was a total number of kidney cancer patients with each medical condition, while the numerator represented the subset of those patients who either had the condition as a single disease or in addition to another comorbid condition. More detailed explanations are described in the result section below. To identify the associations of individual comorbidity with age and sex, multivariable logistic regression models were conducted to estimate the odds ratio (OR) of having each comorbidity (yes vs. no) in ten of the most prevalent conditions. The regression model included age and sex as independent covariates and adjusted for other remaining comorbidities. The OR for the number of comorbidities (1–2, 3–4, and ≥ 5 vs. none) was estimated with age and sex as independent covariates in multinomial logistic regression models. Short‐ and long‐term overall survival probability of kidney cancer patients by year of diagnosis from 2010 to 2021 were estimated by age and sex through Kaplan–Meier curves at different time frames: 3‐month, 6‐month, 1‐year, 3‐year, and 5‐year post‐diagnosis. Kaplan–Meier curves for overall survival by the number of comorbidities were also applied, and further stratified by sex, age groups (20–39, 40–59, 60–79, and ≥ 80 years), and COPD (yes vs. no). All data analyses were conducted using SAS Version 9.4.

## 3 Results

### 3.1 Kidney Cancer Incidence

A total of 42,740 kidney cancer patients were included, and their age and sex stratified by year of diagnosis from 2010 to 2021 are shown in Table 1 and Figure S3. Overall, 68.7% were men, and the median (interquartile range) age was 59 (49–68) years. Incident cases of kidney cancer increased over the recent 12‐year period: 2584 cases (age‐standardized IR 8.0 per 100,000 individuals) in 2010 and 4639 cases (age‐standardized IR 10.7 per 100,000 individuals) in 2021. This increasing trend was observed in both men (age‐standardized IR 11.6 per 100,000 individuals in 2010 and age‐standardized IR 15.3 per 100,000 individuals in 2021) and women (age‐standardized IR 4.9 per 100,000 individuals in 2010 and age‐standardized IR 6.3 per 100,000 individuals in 2021), although the age‐standardized IR in men was over two times higher than in women.

**TABLE 1:** Demographic characteristics as percentage (%) of kidney cancer patients and age‐standardized incidence rate of kidney cancer per 100,000 individuals by sex, age groups, and year of kidney cancer diagnosis.

	2010%	2011%	2012%	2013%	2014%	2015%	2016%	2017%	2018%	2019%	2020%	2021%	Overall%
Total patients, *N* (IR)	2584 (8.0)	2926 (8.7)	2992 (8.7)	3106 (8.7)	3292 (9.0)	3300 (8.8)	3630 (9.4)	3909 (9.9)	3876 (9.6)	4263 (10.3)	4223 (10.0)	4639 (10.7)	42,740
Sex (IR)													
Men	67.7 (11.6)	67.4 (12.6)	68.7 (12.6)	68.5 (12.6)	69.2 (13.1)	67.8 (12.7)	67.1 (13.2)	68.5 (14.2)	69.0 (13.9)	68.5 (14.6)	69.7 (14.4)	69.3 (15.3)	68.5
Women	32.3 (4.9)	32.6 (5.5)	31.4 (5.2)	31.5 (5.3)	30.8 (5.3)	32.2 (5.4)	32.9 (6.0)	31.5 (6.0)	31.0 (5.7)	31.5 (6.2)	30.3 (5.8)	30.7 (6.3)	31.5
Age, median (Q1–Q3) (years)	58 (49–68)	58 (49–68)	57 (49–67)	58 (48–68)	58 (49–68)	59 (50–68)	58 (49–68)	59 (49–68)	59 (50–68)	59 (49–68)	60 (50–68)	60 (50–68)	59 (49–68)
20–39	9.2	9.0	8.5	8.5	8.7	8.1	7.6	8.5	8.1	8.0	8.8	7.4	8.3
40–49	16.9	17.3	17.5	18.6	17.4	15.3	17.6	16.8	16.7	17.2	15.7	17.1	17.0
50–59	29.2	28.9	30.6	28.1	27.8	29.2	27.8	27.2	25.5	26.3	24.9	24.4	27.2
60–69	24.5	23.6	22.5	22.7	24.2	24.8	25.7	25.6	27.4	27.0	28.9	29.9	25.9
70–79	16.9	17.4	16.1	18.2	16.9	17.3	15.9	15.9	16.2	16.1	16.4	15.9	16.5
80+	3.3	3.9	5.0	3.9	5.0	5.3	5.5	5.9	6.1	5.5	5.4	5.3	5.1

Abbreviation: IR, age‐standardized incidence rate per 100,000 individuals.

### 3.2 Comorbidity Prevalence at Kidney Cancer Diagnosis

**FIGURE 1:**
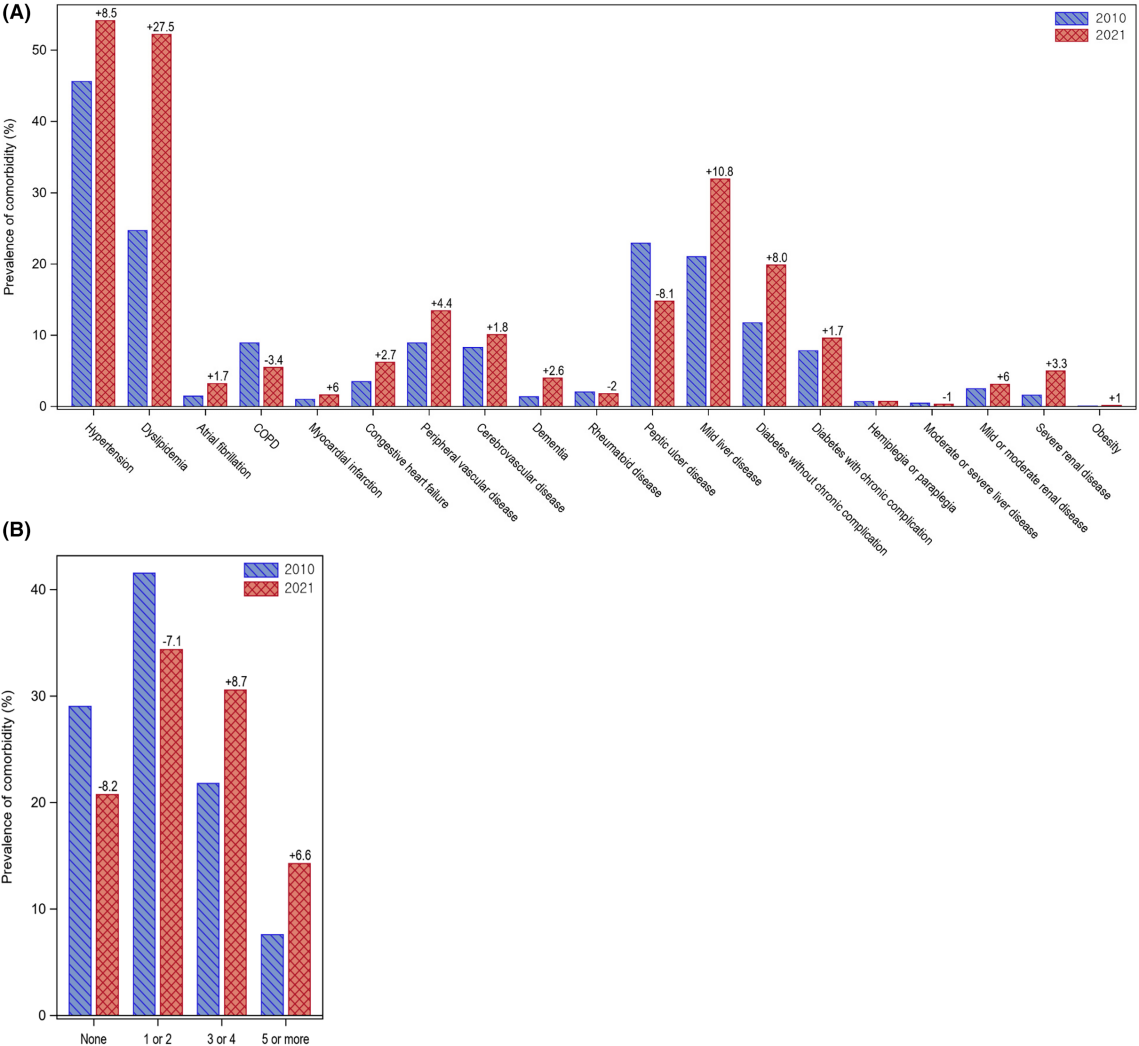
Prevalence (%) of comorbidities and number of comorbidities in 2010 and 2021 among kidney cancer patients. (A) Prevalence (%) of comorbidities in 2010 and 2021 among kidney cancer patients. Most comorbidity prevalences were increased over 12 years, with the exception of COPD, rheumatoid disease, peptic ulcer disease, hemiplegia/paraplegia, and moderate or severe liver disease. (B) Number of comorbidities (%) in 2010 and 2021 among kidney cancer patients. Increasing trends of having more than three multiple comorbidities were observed. The numbers between the two bar plots represent the absolute difference (%) in comorbidity prevalence and number of comorbidities in 2021 compared to 2010. COPD, chronic obstructive pulmonary disease.

Prevalence of individual comorbidity and the number of comorbidities in 2010 and in 2021 is shown in Figure 1. Among the 19 conditions, most comorbidity prevalences have increased when compared to 2010 to 2021, while COPD, rheumatoid disease, peptic ulcer disease, hemiplegia/paraplegia, and moderate or severe liver disease decreased. Approximately 70.9% had one or more comorbidities at the time of kidney cancer diagnosis in 2010, and it increased to 79.2% in 2021 (Table 2). Overall, hypertension (51.3%) and dyslipidemia (40.2%) were the most common comorbidities, followed by mild liver disease (27.4%), diabetes with or without chronic complication (25.1%), and peptic ulcer disease (18.9%). Over the recent 12‐year period, prevalence of hypertension, dyslipidemia, and mild liver disease increased steadily, while prevalence of peptic ulcer disease continuously decreased. Increasing trends of having more than three multiple comorbidities were observed (29.4% in 2010 and 44.9% in 2021). Table 3 shows the prevalence of comorbidity by sex and age groups. The five most prevalent comorbidities, above mentioned, were the same in both men and women, while dyslipidemia (38.8% for men vs. 43.3% for women) and peptic ulcer disease (17.9% vs. 21.3%) were more common in women, and mild liver disease (29.1% vs. 23.6%) was more prevalent in men. As expected, older patients have relatively more comorbidities than younger groups (3 or more comorbidities: 38.0% in kidney cancer patients < 40 years and 62.8% in those over 80 years).

**TABLE 2:** Comorbidity prevalence as percentage of kidney cancer patients by comorbidity type, number of comorbidities, and year of diagnosis.

Comorbidities	2010%	2011%	2012%	2013%	2014%	2015%	2016%	2017%	2018%	2019%	2020%	2021%	Overall%
Total patients, *N*	2584	2926	2992	3106	3292	3300	3630	3909	3876	4263	4223	4639	2740
Hypertension (+)	45.6	47.2	49.4	49.8	49.8	50.9	52.1	50.6	52.7	53.0	54.9	54.2	51.3
Dyslipidemia (+)	24.7	28.5	29.8	32.7	35.5	37.3	41.6	41.8	44.2	46.8	50.3	52.2	40.2
Atrial fibrillation	1.5	1.7	2.0	1.9	1.7	1.9	2.3	2.5	2.8	2.6	3.0	3.2	2.3
COPD	8.9	11.1	8.0	7.6	6.7	8.8	7.6	8.0	7.8	7.0	6.7	5.5	7.6
Myocardial infarction	1.0	1.1	0.8	1.0	1.2	1.2	1.2	1.3	1.4	1.3	1.6	1.6	1.3
Congestive heart failure	3.5	3.6	4.5	4.4	4.0	4.3	5.3	5.5	5.4	5.5	5.8	6.2	5.0
Peripheral vascular disease	8.9	10.0	9.7	9.1	9.5	11.0	11.4	12.2	12.2	12.7	13.3	13.4	11.4
Cerebrovascular disease	8.3	9.0	9.8	8.8	9.3	8.8	9.1	8.5	9.4	9.8	10.0	10.1	9.3
Dementia	1.4	2.0	2.1	2.0	2.8	2.3	2.6	3.5	4.0	3.7	3.4	4.0	2.9
Rheumatoid disease	2.1	2.0	2.2	2.2	2.2	2.4	2.2	2.3	2.1	2.0	1.9	1.8	2.1
Peptic ulcer disease (−)	23.0	22.9	21.8	21.0	21.0	19.8	18.3	18.2	17.5	17.3	16.7	14.8	18.9
Mild liver disease (+)	21.1	24.1	24.0	25.4	25.8	25.4	26.5	28.3	28.5	29.7	31.5	31.9	27.4
Diabetes without chronic complication	11.8	13.3	13.3	14.9	15.2	16.7	16.4	16.5	16.6	18.0	20.5	19.9	16.5
Diabetes with chronic complication	7.8	9.1	8.5	8.1	8.3	7.8	8.5	8.6	9.2	9.1	8.4	9.6	8.6
Hemiplegia or paraplegia	0.7	0.8	0.7	0.6	0.6	0.6	0.5	0.7	0.9	0.7	0.8	0.7	0.7
Moderate or severe liver disease	0.5	0.7	0.2	0.3	0.2	0.4	0.1	0.3	0.2	0.1	0.1	0.3	0.3
Mild or moderate renal disease	2.5	2.1	2.1	2.5	2.2	2.8	2.7	2.6	3.0	2.7	4.0	3.2	2.8
Severe renal disease	1.6	3.3	3.0	3.4	2.9	3.6	3.5	4.2	4.8	4.3	4.2	5.0	3.8
Obesity	0.0	0.0	0.0	0.2	0.0	0.0	0.3	0.1	0.2	0.2	0.1	0.2	0.1
Number of comorbidities													
None (−)	29.1	26.5	26.4	26.2	25.9	24.8	23.8	24.6	22.7	22.6	20.3	20.8	24.1
1 or 2 (−)	41.6	39.6	40.2	39.0	38.7	38.3	37.8	35.7	36.5	35.1	34.8	34.4	37.3
3 or 4 (+)	21.8	24.9	24.3	25.5	25.6	25.9	27.3	27.9	27.8	29.4	30.7	30.6	27.2
5 or more (+)	7.6	9.0	9.1	9.3	9.9	11.0	11.1	11.8	13.0	12.9	14.2	14.3	11.4

*Note:* If the three‐year average of comorbidity prevalence is always higher (lower) than the three‐year average of the period immediately before, the trend was identified as increased (decreased). An increased trend was denoted by (+), a decreased trend by (−).

Abbreviation: COPD, chronic obstructive pulmonary disease.

**TABLE 3:** Comorbidity prevalence as percentage (%) of kidney cancer patients from 2010 to 2021 by sex and age groups.

Comorbidities	Sex		Age groups (years)					
	Men%	Women%	20%–39%	40%–49%	50%–59%	60%–69%	70%–79%	80+%
Total patients, *N*	29,292	13,448	3547	7247	11,633	11,068	7060	2185
Hypertension	51.1	51.5	12.5	28.4	46.7	62.3	75.6	79.6
Dyslipidemia	38.8	43.3	13.3	25.9	38.1	49.8	54.1	48.5
Atrial fibrillation	2.5	1.9	0.3	0.6	1.3	2.6	5.2	6.7
COPD	7.5	7.9	3.0	2.9	5.4	9.1	13.9	15.2
Myocardial infarction	1.5	0.7	0.2	0.5	0.9	1.8	2.1	2.6
Congestive heart failure	4.7	5.5	1.2	1.8	3.1	5.5	9.4	14.1
Peripheral vascular disease	10.6	13.0	1.1	3.9	8.5	15.3	20.0	20.2
Cerebrovascular disease	8.9	10.1	0.9	2.6	5.7	11.1	18.9	24.1
Dementia	2.0	5.0	0	0.1	0.5	2.0	7.6	19.5
Rheumatoid disease	1.5	3.5	0.8	1.5	1.6	2.4	3.3	3.3
Peptic ulcer disease	17.9	21.3	10.4	13.4	17.2	21.4	26.2	24.3
Mild liver disease	29.1	23.6	18.2	24.6	29.2	30.9	27.9	21.6
Diabetes without chronic complication	16.6	16.3	4.1	9.6	15.5	20.2	23.5	22.7
Diabetes with chronic complication	8.6	8.7	1.7	3.9	7.1	11.2	14.7	11.4
Hemiplegia or paraplegia	0.7	0.7	0.1	0.4	0.5	0.7	1.3	1.7
Moderate or severe liver disease	0.3	0.2	0.2	0.3	0.4	0.3	0.3	0.1
Mild or moderate renal disease	3.1	2.0	0.7	1.6	2.0	3.0	4.9	5.8
Severe renal disease	4.1	3.1	2.5	3.7	3.8	4.1	4.0	3.3
Obesity	0.1	0.2	0.2	0.3	0.1	0.1	0.0	0.1
Number of comorbidities								
None	24.0	24.2	59.9	41.2	25.6	13.8	7.8	6.0
1 or 2	38.0	35.6	31.6	39.9	41.2	38.3	31.1	31.1
3 or 4	26.9	28.0	7.2	15.4	25.9	33.6	38.3	38.0
5 or more	11.1	12.2	1.3	3.5	7.3	14.3	22.9	24.8

Abbreviation: COPD, chronic obstructive pulmonary disease.

**FIGURE 2:**
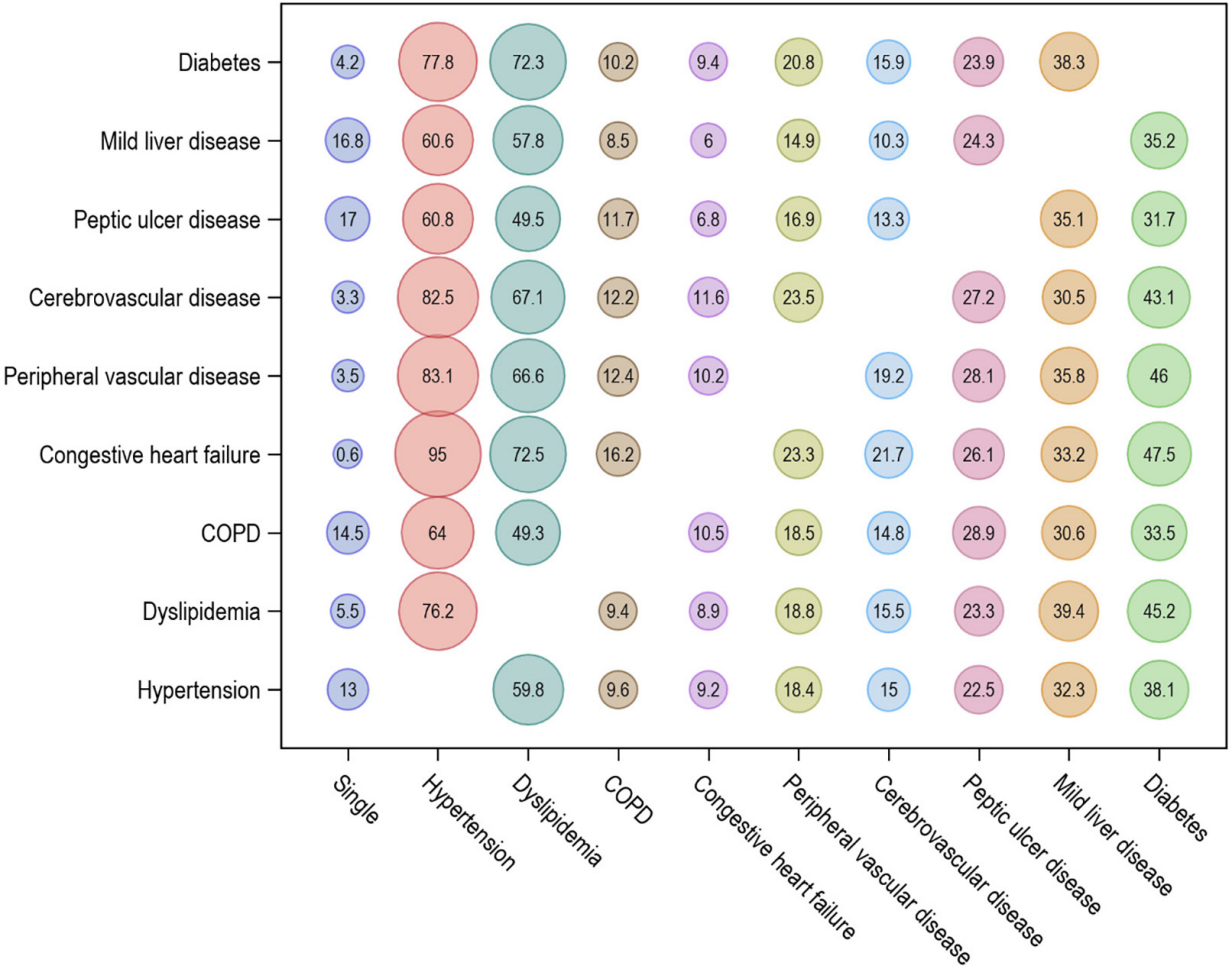
Relative frequency (%) of ten common conditions as a single comorbidity or with another comorbidity among kidney cancer patients. Relative frequency in ten of the most common conditions as the percentage was calculated as follows: the denominator was a total number of patients with the comorbid condition (*y*‐axis), while the numerator represented the subset of those patients who either had the condition as a single disease or in addition to another comorbidity (*x*‐axis). For example, 95.8% of kidney cancer patients with diabetes had additional conditions concurrently (77.8% had hypertension) and only 4.2% of those had diabetes as a single disease. COPD, chronic obstructive pulmonary disease.

Relative frequency of multiple comorbidities is shown in Figure 2. (The *y*‐axis represents the denominator of the relative frequency, and the x‐axis depicts the numerator.) Ten of the most common comorbidities were included: hypertension, dyslipidemia, mild liver disease, peptic ulcer disease, diabetes with or without chronic complications, peripheral vascular disease, cerebrovascular disease, COPD, and congestive heart failure. Hypertension and dyslipidemia were the conditions most commonly present with other comorbidities, ranging from 49.5% to 95.0%. Among kidney cancer patients with congestive heart failure and cerebrovascular disease, 99.4% and 96.7% of individuals had each condition as multiple comorbidities, respectively.

The adjusted ORs of the ten most prevalent comorbidities, previously mentioned, and the number of comorbidities being present at cancer diagnosis by sex and age groups are shown in Table 4 and Figure S4. Compared to women with kidney cancer, men had 41% increased adjusted odds of having mild liver disease (OR = 1.41, 95% confidence interval [CI] 1.34–1.48); 24% increased odds of having hypertension (OR = 1.24, 95% CI: 1.18–1.31); and 18% increased odds of having cerebrovascular disease (OR = 1.18, 95% CI: 1.09–1.28). In contrast, men had 26% decreased adjusted odds of having dyslipidemia (OR = 0.74, 95% CI: 0.71–0.78) and 15% lower odds of having peptic ulcer disease (OR = 0.85, 95% CI: 0.80–0.89). Overall, the odds of having comorbidities at diagnosis consistently increased with age, with the exception of dyslipidemia and mild liver disease. Compared to individuals aged 50–59 years, those over 80 years old had 28% and 42% lower odds of having dyslipidemia and mild liver disease (OR = 0.72, 95% CI: 0.65–0.81 and OR = 0.58, 95% CI: 0.52–0.66, respectively). Kidney cancer patients who were men or older were significantly more likely to have one or more multiple comorbidities compared to others, with the trend being stronger as age increased.

**TABLE 4:** Odds ratios of medical conditions and the number of conditions being present at kidney cancer diagnosis by sex and age groups.

	Comorbidities (yes vs. no)										Number of conditions (vs. none)		
	Hypertension	Dyslipidemia	COPD	CHF	PVD	CVD	PUD	MLD	DM	DMC	1 or 2	3 or 4	5 or more
Sex													
Men	1.24 (1.18–1.31)	0.74 (0.71–0.78)	1.11 (1.02–1.20)	0.86 (0.78–0.96)	0.91 (0.85–0.98)	1.18 (1.09–1.28)	0.85 (0.80–0.89)	1.41 (1.34–1.48)	1.16 (1.10–1.24)	1.13 (1.04–1.23)	1.23 (1.16–1.30)	1.23 (1.16–1.31)	1.28 (1.18–1.39)
Women	Reference	Reference	Reference	Reference	Reference	Reference	Reference	Reference	Reference	Reference	Reference	Reference	Reference
Age groups (years)													
20–39	0.23 (0.21–0.26)	0.51 (0.46–0.58)	0.60 (0.48–0.74)	1.09 (0.77–1.54)	0.22 (0.16–0.31)	0.26 (0.18–0.38)	0.67 (0.59–0.75)	0.76 (0.69–0.84)	0.40 (0.33–0.48)	0.40 (0.30–0.53)	0.33 (0.30–0.36)	0.12 (0.10–0.14)	0.07 (0.05–0.10)
40–49	0.52 (0.49–0.56)	0.82 (0.76–0.88)	0.54 (0.46–0.63)	0.86 (0.701.07)	0.59 (0.52–0.68)	0.56 (0.47–0.66)	0.83 (0.76–0.90)	0.93 (0.87–1.00)	0.71 (0.64–0.79)	0.65 (0.56–0.76)	0.60 (0.56–0.64)	0.37 (0.34–0.40)	0.29 (0.25–0.34)
50–59	Reference	Reference	Reference	Reference	Reference	Reference	Reference	Reference	Reference	Reference	Reference	Reference	Reference
60–69	1.54 (1.45–1.64)	1.18 (1.11–1.26)	1.64 (1.47–1.82)	1.21 (1.05–1.40)	1.52 (1.39–1.65)	1.63 (1.47–1.80)	1.18 (1.10–1.27)	0.93 (0.87–0.99)	1.18 (1.10–1.27)	1.26 (1.14–1.39)	1.75 (1.62–1.88)	2.44 (2.26–2.64)	3.66 (3.30–4.06)
70–79	2.56 (2.38–2.76)	1.00 (0.93–1.07)	2.47 (2.21–2.75)	1.65 (1.43–1.91)	1.73 (1.57–1.90)	2.38 (2.14–2.65)	1.42 (1.31–1.53)	0.75 (0.69–0.80)	1.37 (1.26–1.49)	1.57 (1.41–1.76)	2.54 (2.29–2.82)	4.99 (4.49–5.55)	10.56 (9.33–11.95)
80+	3.37 (2.97–3.82)	0.72 (0.65–0.81)	2.77 (2.38–3.22)	2.58 (2.15–3.10)	1.75 (1.53–2.01)	2.84 (2.46–3.27)	1.26 (1.12–1.42)	0.58 (0.52–0.66)	1.27 (1.12–1.44)	1.11 (0.94–1.33)	3.36 (2.77–4.07)	6.54 (5.40–7.92)	15.16 (12.34–18.62)

Abbreviations: CHF, congestive heart failure; COPD, chronic obstructive pulmonary disease; CVD, cerebrovascular disease; DM, diabetes without chronic complication; DMC, diabetes with chronic complication; MLD, mild liver disease; PUD, peptic ulcer disease; PVD, peripheral vascular disease.

### 3.3 Overall Survival of Kidney Cancer

**FIGURE 3:**
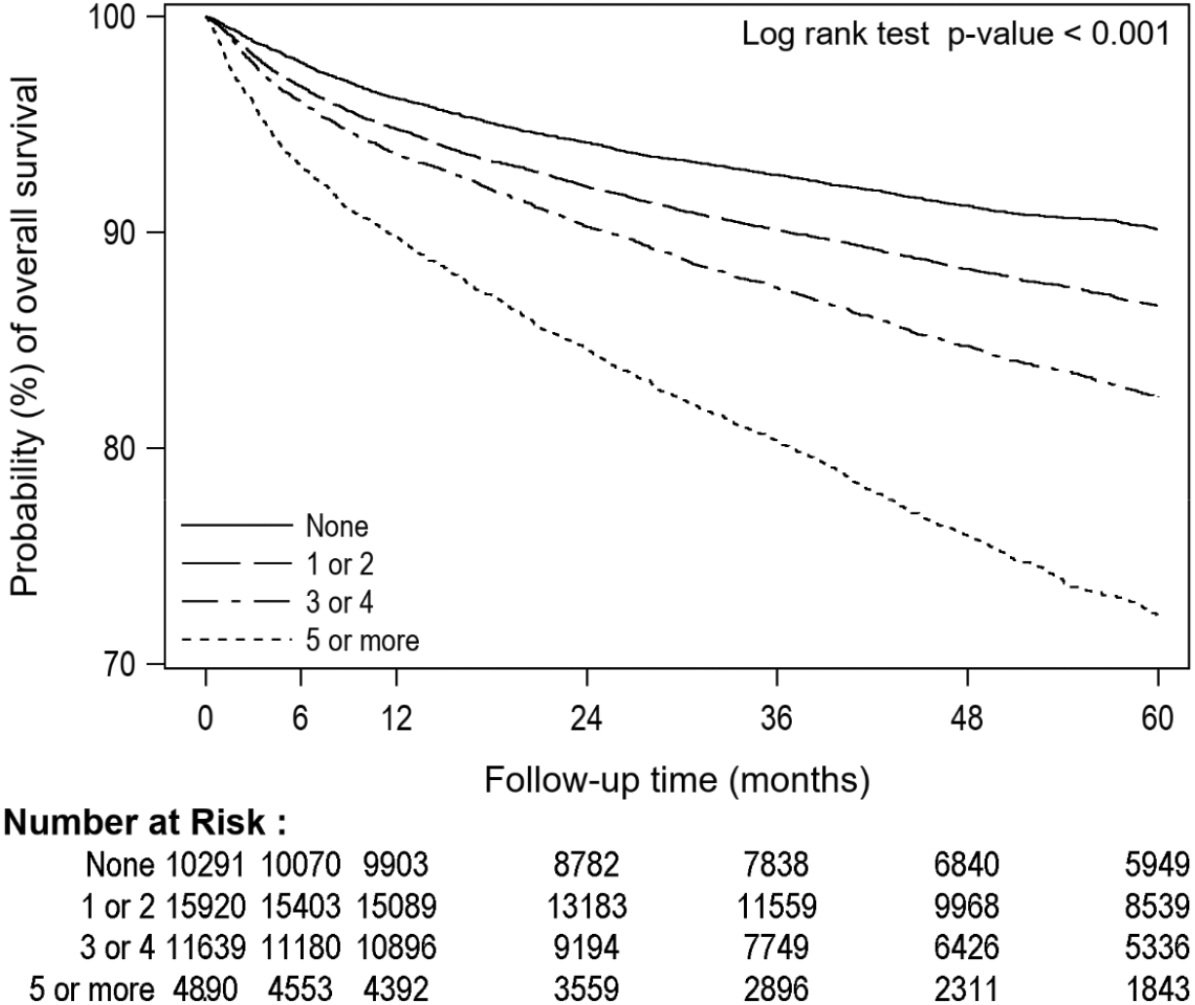
Kaplan–Meier curves for overall survival rate of kidney cancer by the number of comorbidities. The Kaplan–Meier curves by number of comorbidities show decreased trends of survival rates as the number of comorbidities increases among kidney patients.

The probability of overall survival of kidney patients decreased as the number of comorbidities increased (Figure 3). This trend was observed similarly for both men and women (Figure S5A). Kidney cancer patients aged between 40 and 79 years showed a decrease in survival probability with an increasing number of comorbidities, while those aged younger than 40 or older than 80 years showed similar survival rates, regardless of the number of comorbidities (Figure S5B). Table S2 presents the prevalence of each comorbidity among the patients who had 5 or more comorbidities versus those who had 4 or less comorbidities, stratified by age groups. Similar trends were observed in kidney patients, where those with COPD had a lower overall survival probability compared to those without COPD (Figure S5C). Table S3 shows overall survival rates at different time frames for the year of cancer diagnosis (2010–2021) by sex and age groups. Most recent estimations obtained were 85% (5‐year survival rate in 2017), 90% (3‐year in 2019), 95% (1‐year in 2021), 97% (6‐month in 2021), and 98% (3‐month in 2021). Similar survival rates across the time intervals were observed between men and women. The survival rate was lower with increasing age, while the differences by age were more pronounced for longer time windows. The survival rates improved over time in the elderly aged over 80 years, while they remained stable in younger patients.

## 4 Discussion

This nationwide population‐based study, to our knowledge, is the first to provide detailed comorbidity profiles with a longitudinal picture of individual comorbidities of kidney cancer patients in the Asian population. We found that approximately 76% of patients had one or more comorbidities within 1 year prior to kidney cancer diagnosis, and the proportions of patients having multiple comorbid conditions increased over the recent 12‐year period (2010–2021). Hypertension, dyslipidemia, mild liver disease, diabetes, and peptic ulcer disease were the most prevalent comorbidities in kidney cancer patients. Individuals with at least one comorbidity were more likely to have additional comorbidities. Moreover, having more multiple conditions at cancer diagnosis showed a lower probability of overall survival, especially kidney cancer patients aged between 40 and 79 years.

Previous studies have explored a composite measure of comorbidities (e.g., CCI score) in kidney cancer patients, rather than examining individual comorbidities separately. Also, they have not explored their patterns over time. Thus, there are limited existing studies available for direct comparison with our findings regarding the longitudinal prevalence patterns of individual comorbidities. Further, although prior studies have examined the associations between comorbidities and overall survival in kidney cancer patients, there has been limited evidence in Asian populations, on a nationwide scale.

We found that kidney cancer incidence rates have increased over time in the Asian population, consistent with global trends [24], although they remain relatively low compared to the Western population [25]. This rise may be partly explained by our findings of increasing comorbidities, such as hypertension, as well as higher detection and reporting rates [3], an aging population [11, 12], and lifestyle changes [9, 10]. A previous multi‐institutional study conducted in Korea showed that hypertension, diabetes, and mild liver disease were common among kidney cancer patients, thus supporting our findings. However, their prevalence was generally lower compared to our findings [26]. This is likely due to the different sources of the cohorts. While the previous study comprised a highly selected group of patients with non‐metastatic clear cell renal cell carcinoma who underwent radical or partial nephrectomy from several institutions (*N* = 698), the present study included all cases of kidney cancer patients, derived from the entire Korean population. In addition, our study excluded individuals with a history of any cancer, whereas 21.1% of individuals in the previous study had a previous or concurrent cancer. The prevalence of myocardial infarction and moderate or severe liver disease in the previous study was higher than in our study (2.6% vs. 1.3% and 3.3% vs. 0.3%, respectively). This may be because previous or concurrent cancer itself and cancer treatments might contribute to an increased risk of acute myocardial infarction and liver damage [27, 28]. One Danish nationwide study (*N* = 7894) showed that 36% of patients had one or more comorbidities, which is much less than our findings (75.9%) [18]. This is more likely because they only assessed diseases listed in the CCI and did not include hypertension and dyslipidemia. This is supported by our findings that hypertension and dyslipidemia were identified as the most common comorbid conditions and continuously increased over the past 12 years. In addition, we found that approximately half of kidney cancer patients had either hypertension or dyslipidemia, and patients with at least one comorbid condition were more likely to also have hypertension and dyslipidemia. For example, kidney cancer patients with diabetes additionally had hypertension (77.8%) and dyslipidemia (72.3%). A previous US study (*N* = 697) showed that nearly 75% had one or more comorbid conditions, which aligns with our findings [5]. Our study adds to the previous findings by showing the comprehensive prevalence patterns of individual comorbidities over the recent 12‐year period in the Asian population. Kidney cancer patients simultaneously face a high burden of multiple health conditions. Our data emphasizes the importance of multidisciplinary care for both comorbid conditions and cancer throughout cancer survivorship.

Compared to women, men had mostly higher odds of having comorbidities, with the exception of dyslipidemia, heart failure, peripheral vascular disease, and peptic ulcer disease. The odds of having comorbidity generally peaked in the oldest group, aged over 80 years. On the other hand, the odds of having dyslipidemia and mild liver disease showed different trends with age. The prevalence of dyslipidemia rose with the increase in age and peaked at age 60–70 years, and then began to present a downward trend. This trend is supported by the recent statistics in Korea on the distribution of serum lipid concentrations by age, that showed similar trends, as depicted in the 2018 Korean guidelines for the management of dyslipidemia [29]. For mild liver disease, the lowest odds was observed in the group aged over 80, which are in line with previous findings on different cancer sites such as colon, rectal, and lung cancers [30]. This is probably due to the various risk factors for liver disease, other than aging, such as liver cirrhosis or hepatitis, hepatitis B or C virus infections, autoimmune disease, or fatty liver‐related obesity or diabetes [31]. Additionally, the lower odds of some comorbidities in the 80+ age group might reflect survival bias, where individuals with fewer or less severe comorbidities tend to live longer. We acknowledge that this can skew the risk profile for this age group, especially in comparisons across age groups.

Although overall survival increased over time, it was attenuated by having more comorbidities. Our findings are supported by previous studies, regardless of histologic types, stages, primary or secondary cancer, or various metrics of comorbidity in different racial/ethnic populations [5, 6, 18, 26, 32]. As our study population comprised individuals without a history of cancer, our survival probabilities were on average higher than previous estimates. Our data adds to the existing literature by showing that among kidney cancer patients aged between 40 and 79 years, having multiple comorbidities significantly affects lowering their overall survival. Conversely, in patients < 40 or ≥ 80 years, comorbidities do not significantly influence survival outcomes. Our findings suggest that different age groups have varying impacts on how comorbidities affect cancer patient survival. Since cancer stage is pivotal for prognosis, however, further studies with cancer data are needed to explore age‐related disparities in survivorship.

Comorbidity status could impact on when cancer is detected; symptoms associated with comorbid conditions might prompt patients to visit a clinic sooner, potentially resulting in an earlier diagnosis [15, 16]. Conversely, nonspecific symptoms of kidney cancer might be mistakenly considered as symptoms of a pre‐existing disease, such as abdominal or back pain, blood in urine, loss of appetite, tiredness, or fever, potentially causing a delay in diagnosis [15, 16]. Comorbidities could also interfere with initiating or completing a diagnostic evaluation [13, 15]. In addition, comorbidities could contribute to a delay in the cancer treatment decision, initiation, or completion, extension of hospitalization after nephrectomy, worse prognosis, development of treatment‐related complications, or decrease in quality of life or survival [5, 6, 7, 8, 13, 14, 33].

Despite these crucial impacts of comorbidity on cancer patients, most guidelines do not address the simultaneous management of cancer and comorbidity; instead, they focus on managing cancer as a single disease [4, 17]. Furthermore, older cancer patients, who are more likely to have multiple comorbidities, have been underrepresented in clinical trials. This implies that the evidence may not fully apply to older and less healthy cancer patients, potentially resulting in suboptimal care for both cancer and comorbid conditions in real‐world practice. Our findings could serve as the foundational step in delineating comprehensive management strategies tailored to the individual profiles of kidney cancer patients.

Our study has several strengths, including the nationwide population‐based design, a substantial cohort of kidney cancer patients, a recent and long‐term study period, and systematic methodologies for assessing the longitudinal patterns of comorbidity and survival. Several limitations also should be noted. First, we identified comorbidity information based on diagnostic records, which may have measurement errors. However, we believe that chronic or severe conditions requiring treatment or follow‐up, such as hypertension or diabetes, were likely to be adequately captured. In contrast, less severe conditions, such as obesity, might be underestimated. Second, due to a lack of cancer information in the health insurance database, we were unable to investigate the prevalence of comorbidity and its impact on survival according to cancer stage, grade, histology, and treatments. Additionally, since smoking status, a strong risk factor for kidney cancer, was unavailable, COPD as a proxy of smoking was included in the models. Further research is warranted to expand our findings, incorporating detailed cancer and lifestyle information to optimize pre‐existing conditions and to anticipate vulnerabilities to specific complications in cancer care. Lastly, we observed an increase in comorbidities among kidney cancer patients over time. However, this trend may be biased as it does not account for changes in age. Still, our findings highlight that the actual number of cases is crucial for understanding the overall disease burden.

## 5 Conclusion

This nationwide population‐based study found that comorbidities were prevalent in kidney cancer patients and the proportions of patients having multiple comorbidities increased over the recent 12‐year period. Although overall survival increased over time, it was attenuated by having more comorbidities. Our data emphasizes the importance of comprehensive management for both cancer and comorbidity in kidney cancer patients.

## Author Contributions

**Minji Jung:** conceptualization (lead), investigation (lead), methodology (lead), validation (lead), writing – original draft (lead), writing – review and editing (equal). **Eunjung Choo:** data curation (lead), formal analysis (lead), investigation (lead), methodology (equal), software (lead), visualization (lead), writing – review and editing (supporting). **Jinhui Li:** writing – review and editing (supporting). **Zhengyi Deng:** writing – review and editing (supporting). **Marvin E. Langston:** methodology (supporting), project administration (supporting), supervision (supporting), writing – review and editing (lead). **Sukhyang Lee:** conceptualization (equal), funding acquisition (lead), project administration (lead), resources (lead), software (lead), supervision (lead), writing – review and editing (lead). **Benjamin I. Chung:** conceptualization (lead), project administration (lead), supervision (lead), writing – review and editing (lead).

## Conflicts of Interest

The authors declare no conflicts of interest.

## Supporting information


Data S1.

## Data Availability

The study used the Korean Nationwide Health Insurance Database. The authors cannot legally distribute these data, but details on data access can be found here: https://nhiss.nhis.or.kr/bd/ab/bdaba012eng.do
