# Supplementary material for: Longitudinal Trends of Comorbidities and Survival Among Kidney Cancer Patients in Asian Population
Source: Cancer Med. 2024 Nov 19;13(22):e70421. doi: 10.1002/cam4.70421 (PMC11574737; doi:10.1002/cam4.70421)
Supplement: Supplementary file 1 — Data S1. [file CAM4-13-e70421-s001.docx]

Supplementary Table 1. ICD-10 codes used in this study.

| ICD-10-CM Codes | |
| --- | --- |
| Kidney cancer | C64.x |
| Atrial fibrillation | I48.x |
| Hypertension | I10.x–I15.x |
| Dyslipidemia | E78.x |
| Chronic obstructive pulmonary disease | J41.x-J44.x |
| Mild or moderate renal disease | I12.9, I13.0, I13.10, N03.x, N05.x, N18.1, N18.2, N18.3, N18.4, N18.9, Z94.0 |
| Severe renal disease | I12.0, I13.11, I13.2, N18.5, N18.6, N19.x, N25.0, Z49.x, Z99.2 |
| Obesity | E66.x |
| Cerebrovascular disease | G45.x, G46.x, H34.0, I60.x-I69.x |
| Congestive heart failure | I50.x |
| Dementia | F01.x–F03.x, G30.x–G32.x |
| Diabetes without chronic complication | E10.0, E10.1, E10.6, E10.8, E10.9, E11.0, E11.1, E11.6, E11.8, E11.9, E13.0, E13.1, E13.6, E13.8, E13.9 |
| Mild liver disease | B18.x, K70.0-K70.3, K70.9, K71.3-K71.5, K71.7, K73.x, K74.x, K76.0, K76.2-K76.4, K76.8, K76.9, Z94.4 |
| Myocardial infarction | I21.x, I22.x, I25.2 |
| Peripheral vascular disease | I70.x, I71.x, I73.1, I73.8, I73.9, I77.1, I79.0, I79.2, K55.1, K55.8, K55.9, Z95.8, Z95.9 |
| Peptic ulcer disease | K25.x–K28.x, |
| Rheumatologic disease | M05.x, M06.x, M32.x–M34.x M31.5M35.1, M35.3, M36.0 |
| Diabetes with chronic complication | E10.2–E10.5, E11.2–E11.5, E13.2–E13.5, E14.x |
| Hemiplegia or paraplegia | G04.1, G11.4, G80.1, G80.2, G81.x, G82.x, G83.x |
| Moderate or severe liver disease | I85.0x, I86.4, K70.4x, K71.1x, K72.1x, K72.9x, K76.5, K76.6, K76.7 |
| Abbreviations. HIV/AIDS, human immunodeficiency virus infection and acquired immunodeficiency syndrome; ICD, International Classification of Diseases | |

Supplementary Table 2. Prevalence of each comorbidity among the patients who had 5 or more comorbidities versus those who had 4 or less comorbidities, stratified by age groups (20-39, 40-59, 60-79, and 80 or older groups).

| **Comorbidities** | **Age 20-39, N=3,547** | | | | **Age 40-59, N=18,880** | | | | **Age 60-79, N=18,128** | | | | **Age 80+, N=2,185** | | | |
| --- | --- | --- | --- | --- | --- | --- | --- | --- | --- | --- | --- | --- | --- | --- | --- | --- |
|  | **0-4** | | **5+** | | **0-4** | | **5+** | | **0-4** | | **5+** | | **0-4** | | **5+** | |
|  | **N** | **%** | **N** | **%** | **N** | **%** | **N** | **%** | **N** | **%** | **N** | **%** | **N** | **%** | **N** | **%** |
| HTN | 401 | 11.45 | 41 | 91.11 | 6,450 | 36 | 1,042 | 94.21 | 9,134 | 61.17 | 3,096 | 96.84 | 1,216 | 74.01 | 523 | 96.49 |
| DL | 429 | 12.25 | 42 | 93.33 | 5,300 | 30 | 1,012 | 91.5 | 6,439 | 43.13 | 2,896 | 90.58 | 603 | 36.7 | 457 | 84.32 |
| AF | 7 | 0.20 | 2 | 4.44 | 109 | 1 | 82 | 7.41 | 268 | 1.79 | 384 | 12.01 | 51 | 3.1 | 95 | 17.53 |
| COPD | 100 | 2.86 | 7 | 15.56 | 658 | 4 | 181 | 16.37 | 1,213 | 8.12 | 775 | 24.24 | 174 | 10.59 | 159 | 29.34 |
| MI | 2 | 0.06 | 4 | 8.89 | 73 | 0 | 68 | 6.15 | 115 | 0.77 | 226 | 7.07 | 21 | 1.28 | 36 | 6.64 |
| CHF | 24 | 0.69 | 17 | 37.78 | 253 | 1 | 241 | 21.79 | 474 | 3.17 | 801 | 25.05 | 125 | 7.61 | 183 | 33.76 |
| PVD | 27 | 0.77 | 11 | 24.44 | 827 | 5 | 444 | 40.14 | 1,633 | 10.94 | 1,473 | 46.07 | 228 | 13.88 | 214 | 39.48 |
| CeVD | 23 | 0.66 | 7 | 15.56 | 545 | 3 | 303 | 27.4 | 1,326 | 8.88 | 1,240 | 38.79 | 261 | 15.89 | 265 | 48.89 |
| Dementia | 0 | 0 | 0 | 0 | 36 | 0 | 33 | 2.98 | 342 | 2.29 | 420 | 13.14 | 236 | 14.36 | 191 | 35.24 |
| RD | 19 | 0.54 | 10 | 22.22 | 227 | 1 | 67 | 6.06 | 279 | 1.87 | 224 | 7.01 | 31 | 1.89 | 40 | 7.38 |
| PUD | 344 | 9.82 | 24 | 53.33 | 2,425 | 14 | 552 | 49.91 | 2,717 | 18.2 | 1,503 | 47.01 | 294 | 17.89 | 237 | 43.73 |
| MLD | 613 | 17.50 | 34 | 75.56 | 4,430 | 25 | 754 | 68.17 | 3,553 | 23.8 | 1,834 | 57.37 | 228 | 13.88 | 243 | 44.83 |
| DM | 129 | 3.68 | 18 | 40.00 | 2,019 | 11 | 484 | 43.76 | 2,660 | 17.82 | 1,237 | 38.69 | 290 | 17.65 | 205 | 37.82 |
| DMC | 47 | 1.34 | 12 | 26.67 | 727 | 4 | 384 | 34.72 | 1,157 | 7.75 | 1,117 | 34.94 | 109 | 6.63 | 141 | 26.01 |
| H/P | 3 | 0.09 | 2 | 4.44 | 54 | 0 | 40 | 3.62 | 53 | 0.35 | 113 | 3.53 | 10 | 0.61 | 26 | 4.8 |
| MSLD | 6 | 0.17 | 0 | 0 | 46 | 0 | 17 | 1.54 | 31 | 0.21 | 18 | 0.56 | 1 | 0.06 | 2 | 0.37 |
| MRD | 23 | 0.66 | 3 | 6.67 | 226 | 1 | 120 | 10.85 | 305 | 2.04 | 371 | 11.6 | 52 | 3.16 | 75 | 13.84 |
| SRD | 71 | 2.03 | 17 | 37.78 | 438 | 2 | 272 | 24.59 | 304 | 2.04 | 438 | 13.7 | 28 | 1.7 | 45 | 8.3 |
| Obesity | 6 | 0.17 | 1 | 2.22 | 25 | 0 | 9 | 0.81 | 5 | 0.03 | 4 | 0.13 | 0 | 0 | 1 | 0.18 |

Abbreviations. AF, Atrial fibrillation; CeVD, cerebrovascular disease; CHF, congestive heart failure; COPD, chronic obstructive pulmonary disease; CVD, cerebrovascular disease; DL, dyslipidemia; DM, diabetes without chronic complication; DMC, diabetes with chronic complication; H/P, hemiplegia or paraplegia; HTN, hypertension; MLD, mild liver disease; MRD, mild or moderate renal disease; MSLD, moderate or severe liver disease; PUD, peptic ulcer disease; PVD, peripheral vascular disease; RD, rheumatoid disease; SRD, Severe renal disease

Supplementary Table 3. Overall survival probability with 95% confidence interval of kidney cancer by survival time, sex, age groups and year of diagnosis

|  | 2010 | 2011 | 2012 | 2013 | 2014 | 2015 | 2016 | 2017 | 2018 | 2019 | 2020 | 2021 |
| --- | --- | --- | --- | --- | --- | --- | --- | --- | --- | --- | --- | --- |
| 3-month survival | | | | | | | | | | | | |
| Total | 0.97 (0.97-0.98) | 0.97 (0.97-0.98) | 0.98 (0.98-0.99) | 0.98 (0.98-0.99) | 0.98 (0.98-0.99) | 0.98 (0.97-0.98) | 0.98 (0.97-0.98) | 0.98 (0.97-0.98) | 0.98 (0.98-0.99) | 0.98 (0.98-0.99) | 0.98 (0.97-0.98) | 0.98 (0.98-0.98) |
| Sex | | | | | | | | | | | | |
| Men | 0.98 (0.97-0.98) | 0.98 (0.97-0.98) | 0.98 (0.97-0.99) | 0.98 (0.98-0.99) | 0.98 (0.98-0.99) | 0.98 (0.97-0.98) | 0.98 (0.98-0.99) | 0.98 (0.97-0.99) | 0.99 (0.98-0.99) | 0.99 (0.98-0.99) | 0.98 (0.97-0.98) | 0.98 (0.98-0.99) |
| Women | 0.97 (0.96-0.98) | 0.97 (0.96-0.98) | 0.98 (0.97-0.99) | 0.98 (0.97-0.99) | 0.98 (0.97-0.99) | 0.98 (0.97-0.99) | 0.97 (0.96-0.98) | 0.97 (0.96-0.98) | 0.98 (0.97-0.98) | 0.97 (0.97-0.98) | 0.98 (0.97-0.99) | 0.97 (0.97-0.98) |
| Age groups | | | | | | | | | | | | |
| 20-39 | 1.00 (0.99-1.00) | 0.99 (0.98-1.00) | 1.00 (1.00-1.00) | 1.00 (0.99-1.00) | 1.00 (0.99-1.00) | 1.00 (1.00-1.00) | 1.00 (1.00-1.00) | 1.00 (0.99-1.00) | 1.00 (1.00-1.00) | 1.00 (1.00-1.00) | 1.00 (1.00-1.00) | 1.00 (1.00-1.00) |
| 40-49 | 0.99 (0.99-1.00) | 0.99 (0.98-1.00) | 1.00 (0.99-1.00) | 0.99 (0.99-1.00) | 0.99 (0.99-1.00) | 1.00 (0.99-1.00) | 1.00 (0.99-1.00) | 1.00 (1.00-1.00) | 1.00 (0.99-1.00) | 0.99 (0.99-1.00) | 0.99 (0.99-1.00) | 1.00 (0.99-1.00) |
| 50-59 | 0.99 (0.98-1.00) | 0.99 (0.99-1.00) | 0.99 (0.99-1.00) | 0.99 (0.98-1.00) | 1.00 (0.99-1.00) | 0.99 (0.98-0.99) | 0.99 (0.99-1.00) | 0.99 (0.98-1.00) | 1.00 (0.99-1.00) | 0.99 (0.99-1.00) | 0.99 (0.98-0.99) | 0.99 (0.98-1.00) |
| 60-69 | 0.97 (0.96-0.99) | 0.98 (0.97-0.99) | 0.97 (0.96-0.99) | 1.00 (0.99-1.00) | 0.99 (0.98-1.00) | 0.98 (0.98-0.99) | 0.99 (0.98-1.00) | 0.99 (0.98-0.99) | 0.99 (0.99-1.00) | 0.99 (0.99-1.00) | 0.99 (0.98-0.99) | 0.99 (0.98-0.99) |
| 70-79 | 0.95 (0.93-0.97) | 0.95 (0.93-0.97) | 0.97 (0.95-0.98) | 0.95 (0.93-0.97) | 0.96 (0.95-0.98) | 0.96 (0.94-0.98) | 0.96 (0.94-0.98) | 0.96 (0.94-0.98) | 0.96 (0.95-0.98) | 0.97 (0.95-0.98) | 0.95 (0.94-0.97) | 0.97 (0.96-0.98) |
| 80+ | 0.80 (0.71-0.89) | 0.79 (0.71-0.86) | 0.86 (0.80-0.91) | 0.89 (0.84-0.95) | 0.88 (0.83-0.93) | 0.89 (0.84-0.93) | 0.84 (0.78-0.89) | 0.85 (0.80-0.90) | 0.88 (0.84-0.92) | 0.86 (0.82-0.91) | 0.89 (0.85-0.93) | 0.85 (0.81-0.89) |
| 6-month survival | | | | | | | | | | | | |
| Total | 0.96 (0.95-0.97) | 0.96 (0.95-0.96) | 0.96 (0.96-0.97) | 0.97 (0.96-0.97) | 0.97 (0.96-0.97) | 0.96 (0.95-0.97) | 0.96 (0.95-0.97) | 0.96 (0.95-0.97) | 0.97 (0.96-0.97) | 0.97 (0.96-0.97) | 0.97 (0.96-0.97) | 0.97 (0.96-0.97) |
| Sex | | | | | | | | | | | | |
| Men | 0.96 (0.96-0.97) | 0.96 (0.95-0.97) | 0.96 (0.96-0.97) | 0.97 (0.96-0.97) | 0.97 (0.96-0.98) | 0.96 (0.95-0.97) | 0.96 (0.96-0.97) | 0.96 (0.96-0.97) | 0.97 (0.96-0.98) | 0.97 (0.96-0.98) | 0.97 (0.96-0.97) | 0.97 (0.97-0.98) |
| Women | 0.96 (0.94-0.97) | 0.95 (0.94-0.96) | 0.96 (0.95-0.97) | 0.96 (0.95-0.98) | 0.96 (0.95-0.98) | 0.96 (0.95-0.97) | 0.95 (0.94-0.96) | 0.95 (0.94-0.96) | 0.96 (0.95-0.97) | 0.96 (0.95-0.97) | 0.96 (0.95-0.97) | 0.96 (0.95-0.97) |
| Age groups | | | | | | | | | | | | |
| 20-39 | 0.99 (0.97-1.00) | 0.99 (0.98-1.00) | 1.00 (1.00-1.00) | 1.00 (0.99-1.00) | 0.99 (0.98-1.00) | 1.00 (0.99-1.00) | 1.00 (1.00-1.00) | 0.99 (0.99-1.00) | 1.00 (1.00-1.00) | 1.00 (1.00-1.00) | 1.00 (0.99-1.00) | 1.00 (1.00-1.00) |
| 40-49 | 0.99 (0.98-1.00) | 0.99 (0.98-1.00) | 1.00 (0.99-1.00) | 0.99 (0.98-1.00) | 0.98 (0.97-0.99) | 0.99 (0.98-1.00) | 0.99 (0.98-1.00) | 0.99 (0.98-1.00) | 0.99 (0.98-1.00) | 0.99 (0.98-1.00) | 0.99 (0.99-1.00) | 1.00 (0.99-1.00) |
| 50-59 | 0.99 (0.98-0.99) | 0.98 (0.98-0.99) | 0.98 (0.98-0.99) | 0.98 (0.97-0.99) | 0.99 (0.98-0.99) | 0.97 (0.96-0.98) | 0.99 (0.98-0.99) | 0.98 (0.97-0.99) | 0.99 (0.99-1.00) | 0.98 (0.97-0.99) | 0.98 (0.97-0.99) | 0.98 (0.97-0.99) |
| 60-69 | 0.96 (0.95-0.98) | 0.96 (0.95-0.98) | 0.96 (0.95-0.98) | 0.98 (0.97-0.99) | 0.98 (0.97-0.99) | 0.97 (0.96-0.98) | 0.97 (0.96-0.98) | 0.97 (0.96-0.98) | 0.98 (0.97-0.99) | 0.98 (0.97-0.99) | 0.98 (0.97-0.98) | 0.98 (0.97-0.98) |
| 70-79 | 0.92 (0.90-0.95) | 0.91 (0.89-0.94) | 0.93 (0.91-0.95) | 0.92 (0.90-0.94) | 0.94 (0.92-0.96) | 0.94 (0.91-0.96) | 0.93 (0.90-0.95) | 0.93 (0.91-0.95) | 0.93 (0.91-0.95) | 0.94 (0.93-0.96) | 0.93 (0.92-0.95) | 0.95 (0.94-0.97) |
| 80+ | 0.72 (0.62-0.81) | 0.68 (0.59-0.76) | 0.77 (0.70-0.84) | 0.84 (0.78-0.91) | 0.81 (0.75-0.87) | 0.77 (0.70-0.83) | 0.72 (0.65-0.78) | 0.78 (0.73-0.83) | 0.80 (0.75-0.85) | 0.76 (0.71-0.82) | 0.82 (0.77-0.87) | 0.79 (0.74-0.84) |
| 1-year survival | | | | | | | | | | | | |
| Total | 0.94 (0.93-0.95) | 0.92 (0.92-0.93) | 0.94 (0.93-0.95) | 0.94 (0.93-0.95) | 0.94 (0.93-0.95) | 0.94 (0.93-0.95) | 0.94 (0.93-0.95) | 0.94 (0.93-0.95) | 0.95 (0.94-0.95) | 0.95 (0.94-0.95) | 0.95 (0.94-0.96) | - |
| Sex | | | | | | | | | | | | |
| Men | 0.95 (0.94-0.96) | 0.92 (0.91-0.94) | 0.94 (0.93-0.95) | 0.94 (0.93-0.95) | 0.94 (0.93-0.95) | 0.94 (0.93-0.95) | 0.94 (0.93-0.95) | 0.94 (0.93-0.95) | 0.95 (0.94-0.96) | 0.95 (0.94-0.96) | 0.95 (0.94-0.96) | - |
| Women | 0.93 (0.91-0.95) | 0.92 (0.91-0.94) | 0.94 (0.93-0.96) | 0.93 (0.92-0.95) | 0.94 (0.93-0.96) | 0.94 (0.93-0.96) | 0.93 (0.92-0.94) | 0.93 (0.91-0.94) | 0.94 (0.93-0.95) | 0.94 (0.93-0.95) | 0.95 (0.94-0.96) | - |
| Age groups | | | | | | | | | | | | |
| 20-39 | 0.99 (0.97-1.00) | 0.98 (0.97-1.00) | 1.00 (1.00-1.00) | 0.98 (0.97-1.00) | 0.98 (0.97-1.00) | 0.99 (0.98-1.00) | 1.00 (0.99-1.00) | 0.99 (0.98-1.00) | 0.99 (0.97-1.00) | 1.00 (0.99-1.00) | 0.99 (0.99-1.00) | - |
| 40-49 | 0.98 (0.97-0.99) | 0.97 (0.96-0.99) | 0.99 (0.98-1.00) | 0.97 (0.95-0.98) | 0.97 (0.95-0.98) | 0.98 (0.97-1.00) | 0.98 (0.97-0.99) | 0.98 (0.97-0.99) | 0.98 (0.97-0.99) | 0.98 (0.97-0.99) | 0.99 (0.98-1.00) | - |
| 50-59 | 0.97 (0.96-0.98) | 0.96 (0.95-0.97) | 0.97 (0.96-0.98) | 0.97 (0.95-0.98) | 0.97 (0.96-0.98) | 0.96 (0.95-0.97) | 0.97 (0.96-0.98) | 0.97 (0.96-0.98) | 0.98 (0.97-0.99) | 0.97 (0.96-0.98) | 0.97 (0.96-0.98) | - |
| 60-69 | 0.93 (0.91-0.95) | 0.93 (0.91-0.95) | 0.94 (0.92-0.95) | 0.95 (0.93-0.96) | 0.96 (0.94-0.97) | 0.95 (0.94-0.97) | 0.95 (0.93-0.96) | 0.94 (0.93-0.96) | 0.96 (0.94-0.97) | 0.96 (0.94-0.97) | 0.96 (0.95-0.97) | - |
| 70-79 | 0.89 (0.86-0.92) | 0.86 (0.83-0.89) | 0.89 (0.86-0.91) | 0.88 (0.86-0.91) | 0.90 (0.87-0.92) | 0.89 (0.86-0.92) | 0.89 (0.87-0.92) | 0.89 (0.87-0.92) | 0.89 (0.87-0.92) | 0.92 (0.90-0.94) | 0.90 (0.88-0.92) | - |
| 80+ | 0.61 (0.51-0.72) | 0.55 (0.46-0.64) | 0.71 (0.64-0.78) | 0.78 (0.71-0.85) | 0.73 (0.66-0.80) | 0.69 (0.63-0.76) | 0.65 (0.58-0.71) | 0.71 (0.65-0.77) | 0.74 (0.69-0.80) | 0.69 (0.63-0.75) | 0.75 (0.69-0.81) | - |
| 3-year survival | | | | | | | | | | | | |
| Total | 0.88 (0.86-0.89) | 0.87 (0.86-0.88) | 0.89 (0.87-0.90) | 0.88 (0.86-0.89) | 0.89 (0.88-0.90) | 0.88 (0.87-0.89) | 0.89 (0.88-0.90) | 0.89 (0.88-0.90) | 0.90 (0.89-0.91) | 0.90 (0.89-0.91) | - | - |
| Sex | | | | | | | | | | | | |
| Men | 0.88 (0.86-0.89) | 0.86 (0.85-0.88) | 0.88 (0.87-0.90) | 0.87 (0.86-0.89) | 0.89 (0.87-0.90) | 0.88 (0.86-0.89) | 0.89 (0.88-0.91) | 0.89 (0.88-0.91) | 0.89 (0.88-0.91) | 0.90 (0.89-0.91) | - | - |
| Women | 0.88 (0.85-0.90) | 0.88 (0.86-0.90) | 0.89 (0.87-0.91) | 0.88 (0.86-0.90) | 0.89 (0.87-0.91) | 0.90 (0.88-0.91) | 0.87 (0.86-0.89) | 0.89 (0.87-0.90) | 0.90 (0.88-0.92) | 0.90 (0.89-0.92) | - | - |
| Age groups | | | | | | | | | | | | |
| 20-39 | 0.97 (0.94-0.99) | 0.97 (0.95-0.99) | 0.96 (0.94-0.99) | 0.96 (0.93-0.98) | 0.98 (0.96-1.00) | 0.98 (0.96-1.00) | 0.98 (0.96-1.00) | 0.98 (0.97-1.00) | 0.97 (0.95-0.99) | 0.97 (0.96-0.99) | - | - |
| 40-49 | 0.95 (0.93-0.97) | 0.93 (0.91-0.95) | 0.96 (0.95-0.98) | 0.94 (0.92-0.96) | 0.94 (0.92-0.96) | 0.96 (0.95-0.98) | 0.97 (0.95-0.98) | 0.96 (0.95-0.98) | 0.97 (0.96-0.98) | 0.96 (0.95-0.98) | - | - |
| 50-59 | 0.92 (0.90-0.94) | 0.93 (0.91-0.94) | 0.94 (0.93-0.96) | 0.92 (0.90-0.94) | 0.92 (0.90-0.94) | 0.93 (0.91-0.94) | 0.94 (0.92-0.95) | 0.94 (0.93-0.96) | 0.96 (0.94-0.97) | 0.94 (0.92-0.95) | - | - |
| 60-69 | 0.87 (0.85-0.90) | 0.86 (0.84-0.89) | 0.88 (0.85-0.90) | 0.88 (0.86-0.90) | 0.91 (0.89-0.93) | 0.90 (0.88-0.92) | 0.90 (0.88-0.92) | 0.88 (0.86-0.90) | 0.91 (0.89-0.92) | 0.92 (0.90-0.93) | - | - |
| 70-79 | 0.76 (0.72-0.80) | 0.77 (0.74-0.81) | 0.77 (0.73-0.81) | 0.76 (0.72-0.79) | 0.80 (0.77-0.83) | 0.79 (0.76-0.82) | 0.79 (0.75-0.82) | 0.82 (0.79-0.85) | 0.79 (0.76-0.82) | 0.84 (0.81-0.86) | - | - |
| 80+ | 0.48 (0.38-0.59) | 0.41 (0.32-0.50) | 0.54 (0.46-0.62) | 0.57 (0.49-0.66) | 0.59 (0.52-0.67) | 0.48 (0.41-0.56) | 0.49 (0.42-0.56) | 0.55 (0.48-0.61) | 0.57 (0.50-0.63) | 0.58 (0.52-0.65) | - | - |
| 5-year survival | | | | | | | | | | | | |
| Total | 0.84 (0.82-0.85) | 0.82 (0.81-0.84) | 0.85 (0.83-0.86) | 0.84 (0.83-0.85) | 0.85 (0.83-0.86) | 0.84 (0.83-0.85) | 0.85 (0.84-0.86) | 0.85 (0.84-0.86) | - | - | - | - |
| Sex | | | | | | | | | | | | |
| Men | 0.83 (0.82-0.85) | 0.81 (0.80-0.83) | 0.84 (0.82-0.86) | 0.84 (0.82-0.85) | 0.84 (0.83-0.86) | 0.82 (0.81-0.84) | 0.86 (0.84-0.87) | 0.85 (0.84-0.87) | - | - | - | - |
| Women | 0.84 (0.82-0.87) | 0.84 (0.82-0.87) | 0.86 (0.84-0.88) | 0.84 (0.82-0.87) | 0.85 (0.83-0.87) | 0.87 (0.85-0.89) | 0.83 (0.81-0.86) | 0.85 (0.83-0.87) | - | - | - | - |
| Age groups | | | | | | | | | | | | |
| 20-39 | 0.96 (0.93-0.98) | 0.96 (0.94-0.99) | 0.96 (0.93-0.98) | 0.95 (0.93-0.98) | 0.98 (0.96-0.99) | 0.98 (0.96-1.00) | 0.96 (0.94-0.99) | 0.98 (0.96-0.99) | - | - | - | - |
| 40-49 | 0.92 (0.90-0.95) | 0.90 (0.88-0.93) | 0.93 (0.91-0.96) | 0.92 (0.90-0.95) | 0.92 (0.90-0.94) | 0.94 (0.92-0.96) | 0.95 (0.94-0.97) | 0.95 (0.93-0.96) | - | - | - | - |
| 50-59 | 0.89 (0.87-0.91) | 0.91 (0.89-0.93) | 0.91 (0.90-0.93) | 0.89 (0.87-0.91) | 0.90 (0.88-0.92) | 0.89 (0.87-0.91) | 0.91 (0.89-0.93) | 0.92 (0.91-0.94) | - | - | - | - |
| 60-69 | 0.84 (0.81-0.86) | 0.81 (0.78-0.84) | 0.85 (0.82-0.87) | 0.86 (0.83-0.88) | 0.86 (0.84-0.88) | 0.85 (0.82-0.87) | 0.87 (0.85-0.89) | 0.84 (0.82-0.86) | - | - | - | - |
| 70-79 | 0.67 (0.63-0.72) | 0.67 (0.63-0.71) | 0.68 (0.64-0.73) | 0.67 (0.64-0.71) | 0.71 (0.67-0.75) | 0.71 (0.67-0.75) | 0.69 (0.66-0.73) | 0.76 (0.72-0.79) | - | - | - | - |
| 80+ | 0.36 (0.26-0.47) | 0.30 (0.21-0.38) | 0.45 (0.37-0.53) | 0.46 (0.37-0.55) | 0.46 (0.38-0.53) | 0.40 (0.33-0.48) | 0.41 (0.34-0.47) | 0.42 (0.35-0.48) | - | - | - | - |

Supplementary Figure 1. Study design and scheme


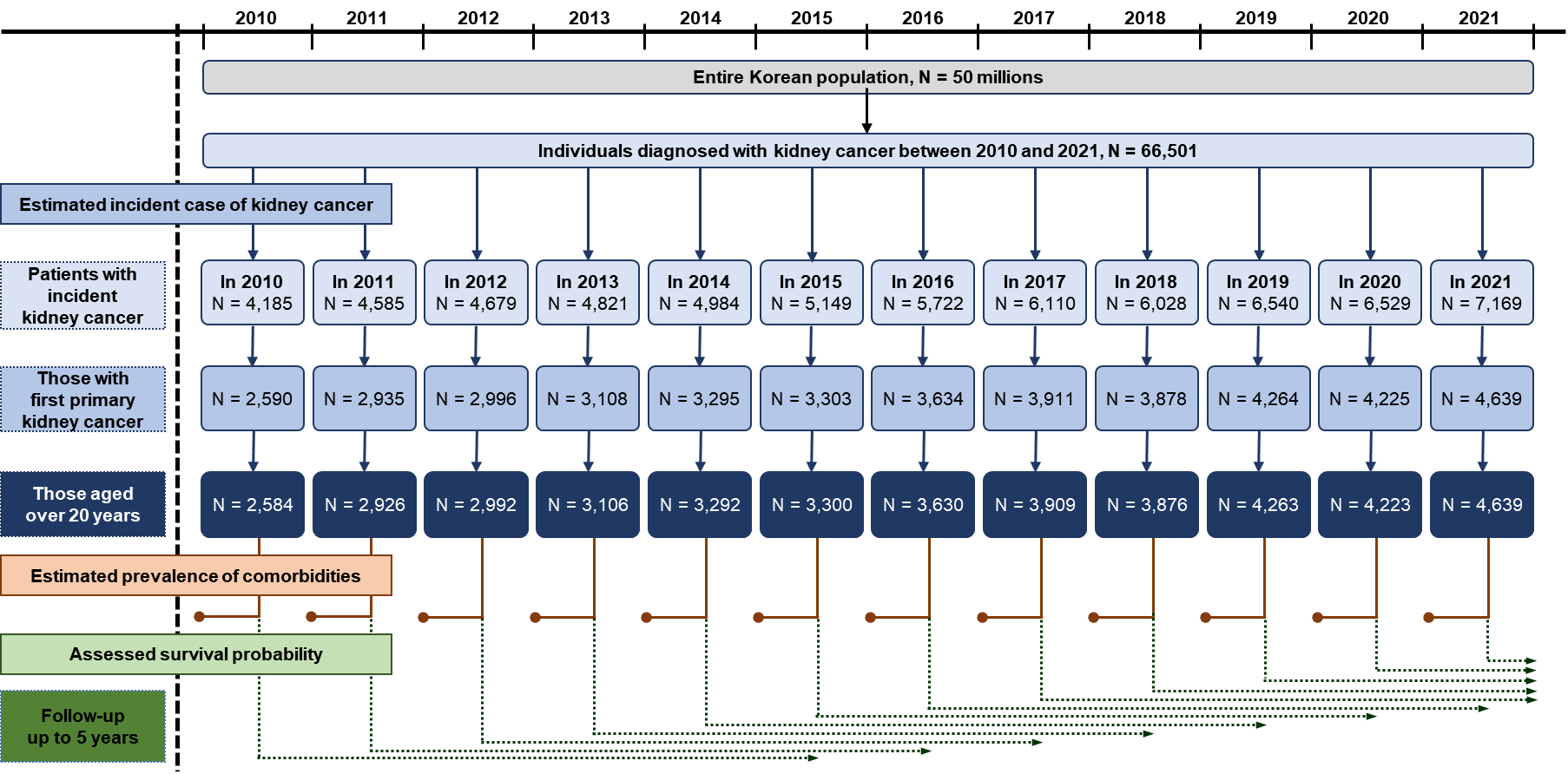


Supplementary Figure 1 Legend.

From the entire Korean population, individuals who were diagnosed with kidney cancer between 2010 and 2021 were identified. We estimated the incidence of kidney cancer cases, prevalence of comorbidities, and probability of survival up to 5 years.

Supplementary Figure 2. Flowchart

Entire Korean population (2007-2021)

N = 50 million

Individuals with a diagnosis of kidney cancer
(2010-2021)

N = 66,501

Kidney cancer survivors

N = 42,740

Excluding:

1. History of any cancers (N = 23,723)
2. Aged younger than 20 years old (N = 38)

Supplementary Figure 2 Legend.

Among the entire Korean population, individuals diagnosed with kidney cancer between 2010 and 2021 were identified. Among these, those aged younger than 18 years or with a history of any cancers, except for non-melanoma skin cancer, before kidney cancer diagnosis were excluded.

Supplementary Figure 3. Demographic characteristics as number of kidney cancer patients by sex, age groups, and year of diagnosis

3A. By sex and year of diagnosis


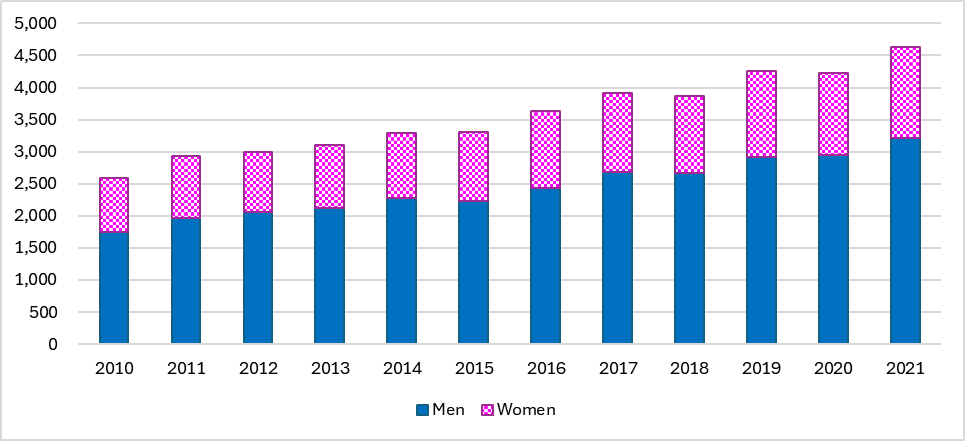


3B. By age groups and year of diagnosis


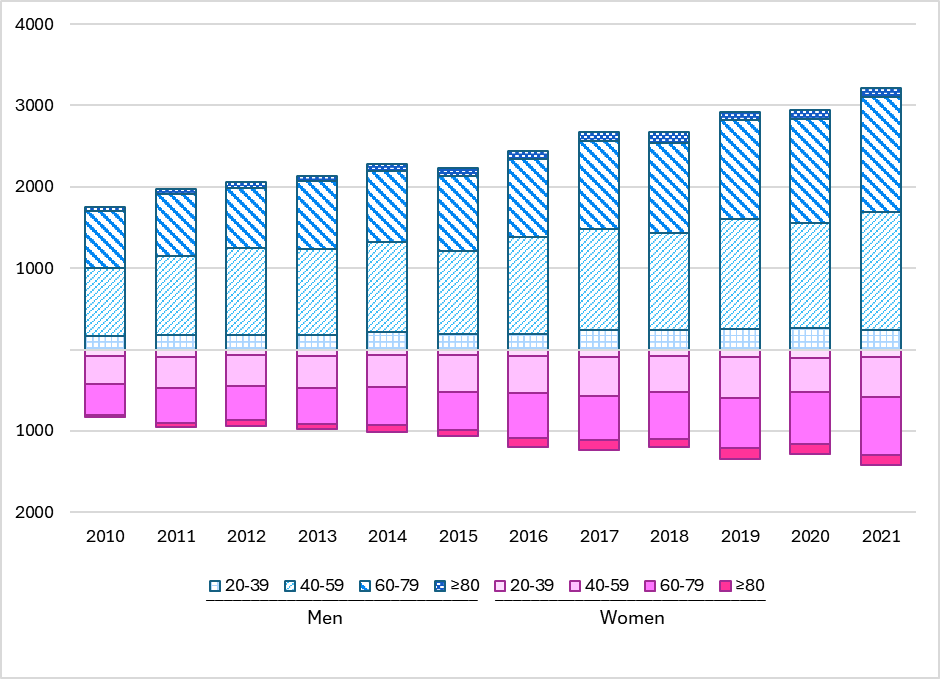


Supplementary Figure 3 Legend.

(3A) Incident cases of kidney cancer increased in both men and women over the recent 12-year period, although the incidence in men was over two times higher than in women. (3B) All incident cases of kidney cancer in different age groups increased over time, and those who were between 40-79 years old were the majority.

Supplementary Figure 4. Odds ratios of medical conditions being present at kidney cancer diagnosis by sex and age groups


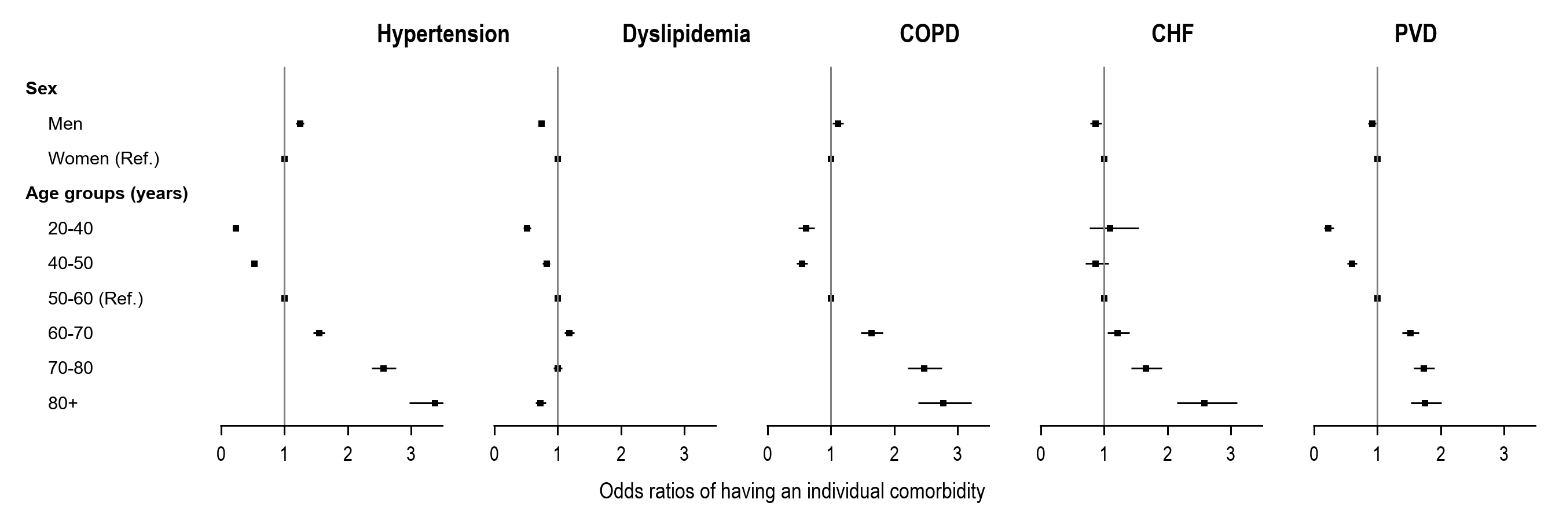


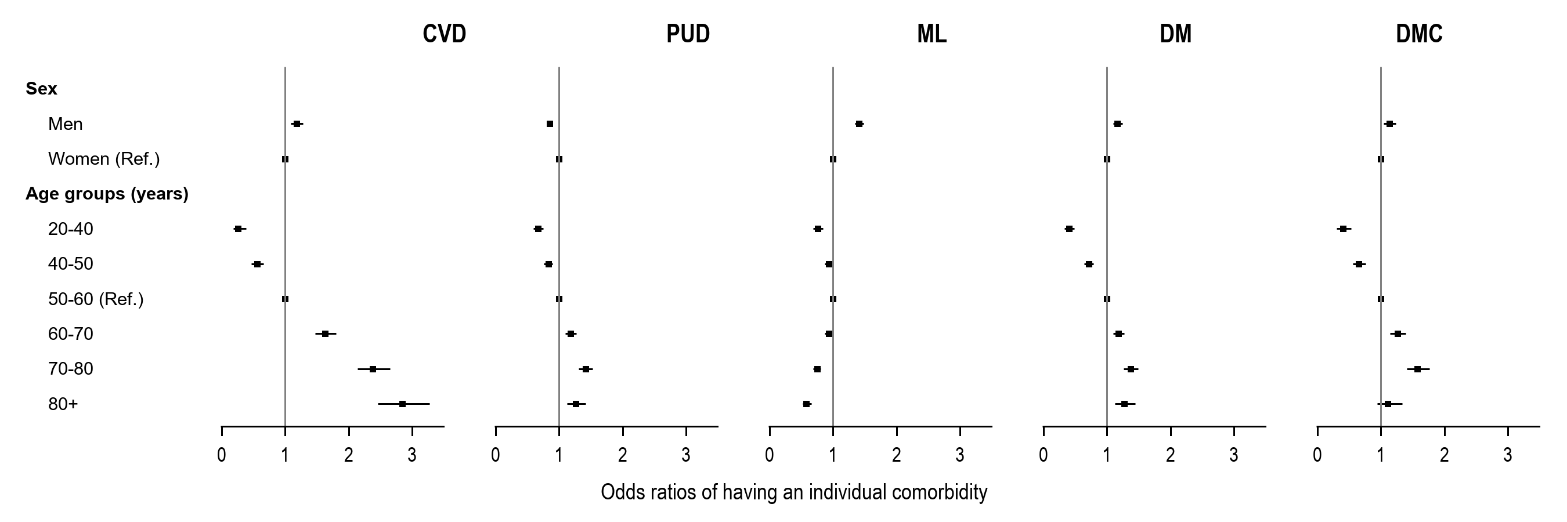


Supplementary Figure 4 Legend.

The odds of having each comorbidity at diagnosis varied according to sex, and generally increased with age, with the exception of dyslipidemia and mild liver disease.

Supplementary Figure 5. Kaplan-Meier curves for overall survival rate of kidney cancer by the number of comorbidities, stratified by sex, age, and COPD groups

5A. By sex (left: men, right: women)


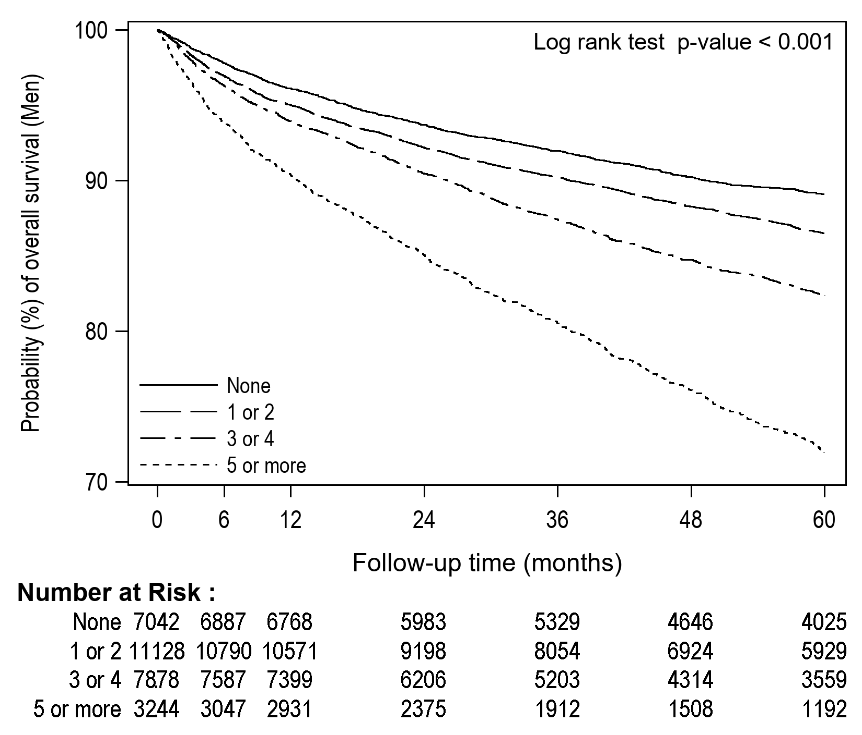

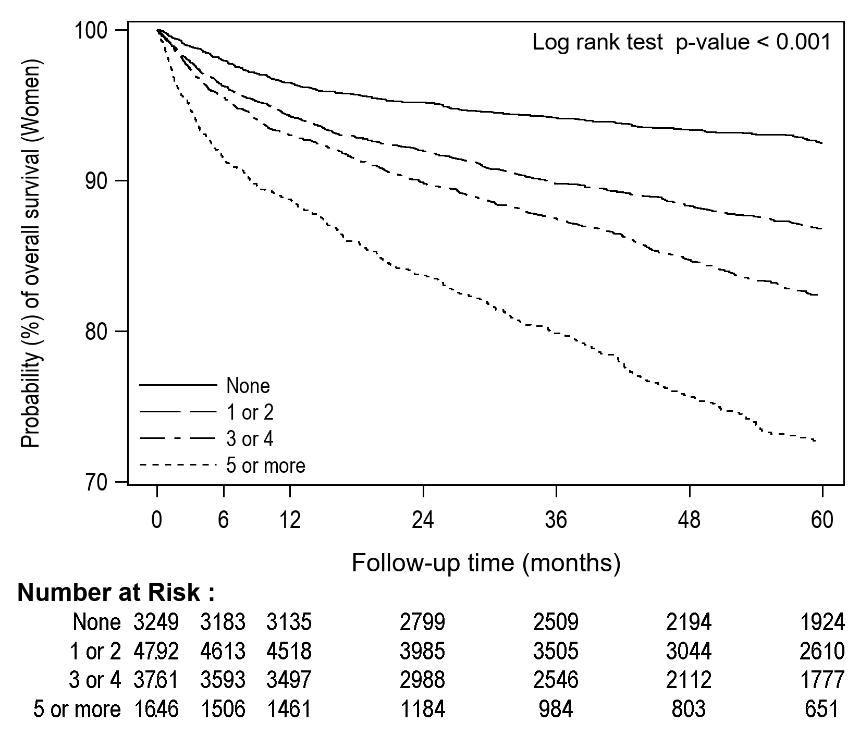


5B. By age groups (left top: age 20-39, right top: 40-59, left bottom: 60-79, right bottom: over 80 years)


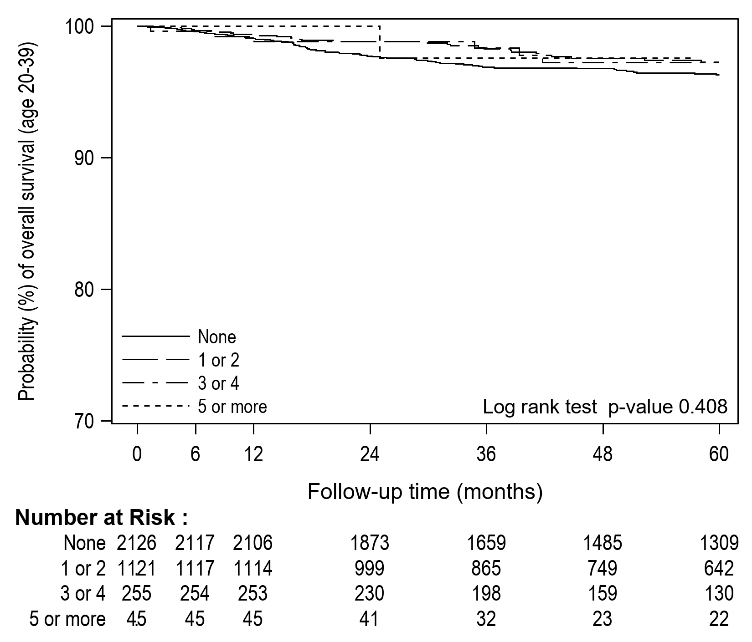

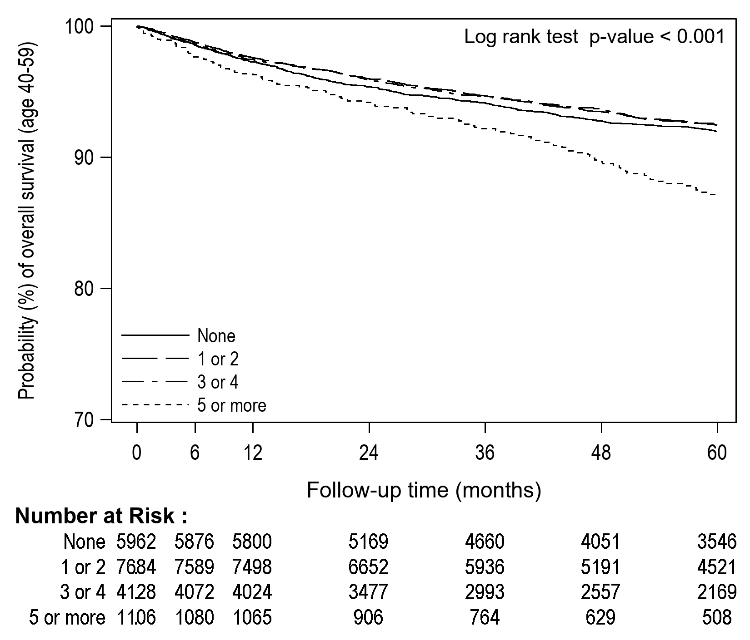


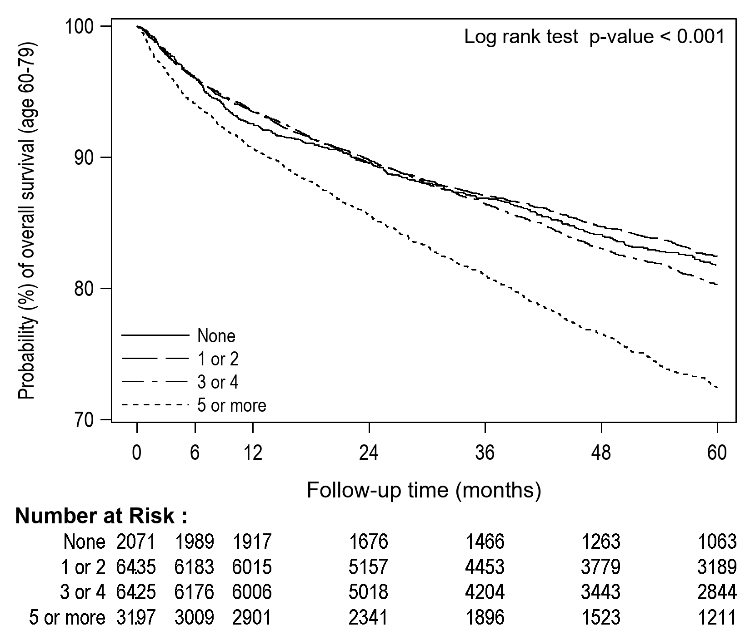

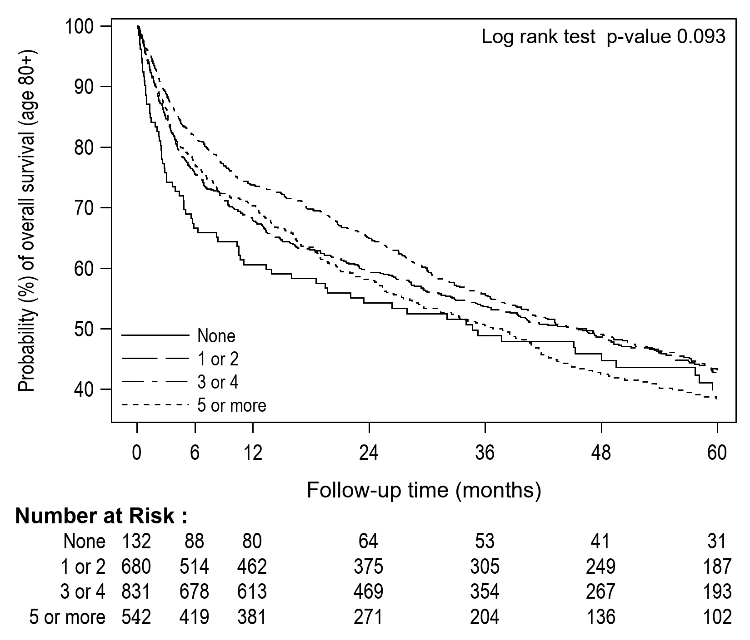


5C. By COPD (left: individuals without COPD, right: individuals with COPD)


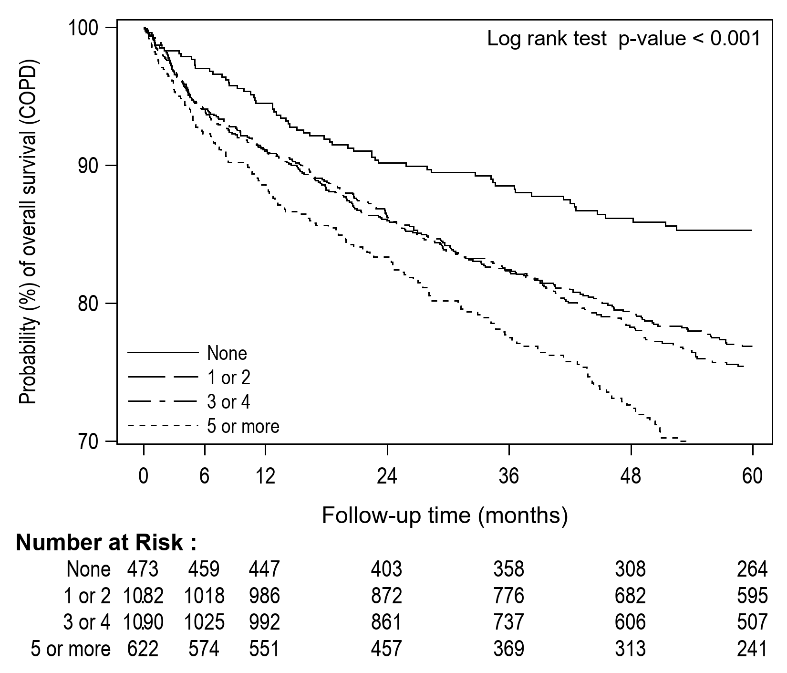

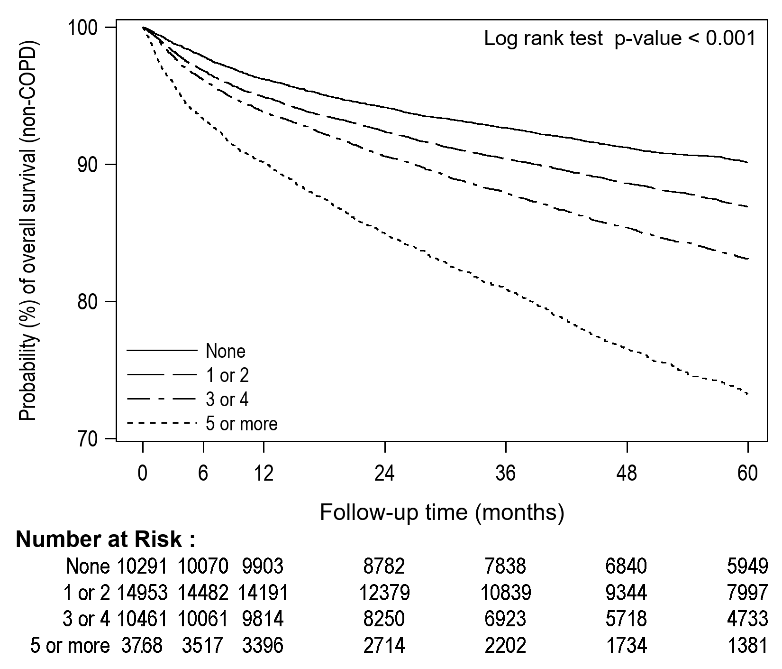


Supplementary Figure 5 Legend.

(5A) The probability of overall survival of kidney patients decreased as the number of comorbidities increased. Both men and women showed similar trends of survival rates. (5B) Kidney patients aged between 40 and 79 years showed a decrease in survival probability with an increasing number of comorbidities, while those aged younger than 40 or older than 80 years showed similar survival rates, regardless of the number of comorbidities. (5C) Overall, similar trends were observed. Kidney patients with COPD showed a lower overall survival probability compared to those without COPD. In this case, the number of comorbid conditions were counted except for COPD.

Abbreviations. COPD, chronic obstructive pulmonary disease
